# One size does not fit all: Lysosomes exist in biochemically and functionally distinct states

**DOI:** 10.1371/journal.pbio.3002576

**Published:** 2024-03-22

**Authors:** Claudio Bussi, Maximiliano G. Gutierrez

**Affiliations:** The Francis Crick Institute, London, United Kingdom

## Abstract

Single-organelle resolution approaches have the potential to advance our knowledge of the heterogeneity of lysosome function. In this Perspective article, the authors propose a ’lysosome states’ concept that links single lysosomes to function.

## Teaser

Single-organelle resolution approaches have the potential to advance our knowledge of the heterogeneity of lysosome function. Challenging population-based models, we propose a “lysosome states” concept that links single lysosomes to function.

Lysosome heterogeneity at the intracellular level is well documented and related to many factors. The positioning of a lysosome within the cell is not random; it is strategic for lysosomal function. For example, perinuclear lysosomes are often involved in degradation, whereas peripheral lysosomes are involved in plasma membrane repair [1]. The size of a lysosome can also differ, influenced by factors such as cellular metabolic needs or external stimuli [2]. Shape is important, and tubular lysosomes have been implicated in a wide range of cellular functions. Adding to this complexity, proteolytic activity and ion concentration have crucial roles in shaping lysosome heterogeneity [1,2]. Biochemical differences in individual lysosomes will also affect the outcomes after damage and leakage of contents. In fact, there is compelling evidence that not all lysosomes undergo damage and/or repair, which suggests the presence of an intrinsic factor that impacts membrane stability [3]. The diversity of lysosome biochemical properties indicates a range of functions beyond degradation, from antigen presentation to cell death regulation [1], and poses a critical challenge: can we correlate these distinct states with specific functions at the individual lysosome level?

To effectively tackle the challenge of lysosome heterogeneity and its correlation with specific cellular functions, we propose the adoption of a “lysosome states” framework. This approach advocates for a detailed classification of lysosomes on the basis of their molecular signature, functional capabilities, localization, and morphological characteristics, each considered at the individual organelle level. By capturing the unique features of each individual lysosome within a larger interconnected network, this framework transcends the limitations of a “one size fits all” model of lysosome function and dynamics.

Most biochemical and cellular characterizations of lysosomes have been conducted using standard tumor-derived cell lines. However, significant differences already exist among these cell lines [4], and even more pronounced distinctions emerge when comparing them to primary differentiated cells [5,6]. Data from our group and others show that the endolysosomal system in these standard cell line models differs from that in differentiated cells [5,6] and in proliferating but non-tumor-derived cells, such as stem cells [7].

These cell type-dependent variations jeopardize broad generalizations of lysosome function and dynamics, emphasizing the importance of considering the diversity inherent in different cell types for a more comprehensive understanding of lysosome biology. Although expanding the range of cell models used in lysosome research would indeed be beneficial, a more critical adjustment could be useful. Our primary focus should shift towards developing methodologies that can accurately account for this diversity. This approach would enable a more precise understanding of lysosome function and dynamics, reflective of the complex biological reality, thereby enhancing the reliability and applicability of lysosome studies in advancing cellular and molecular biology.

**Fig 1:**
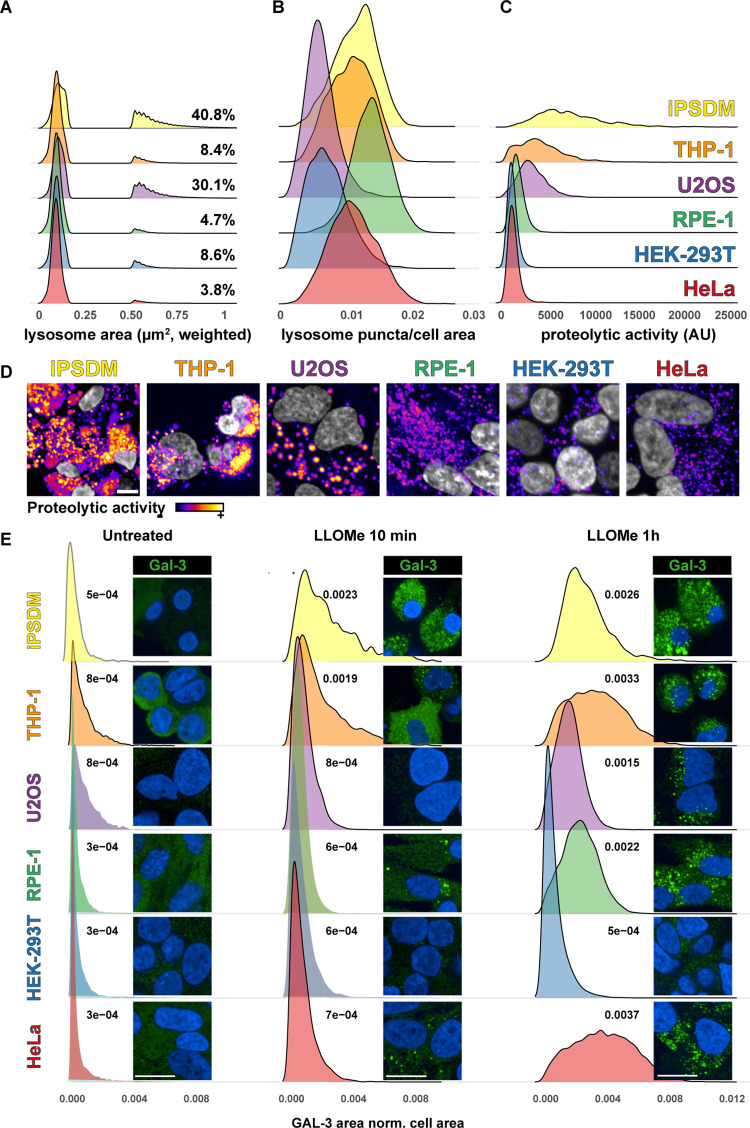
Single lysosome properties across different cell types. **(A)** Lysosome size distribution (quantified as the area based on LysoTracker-positive puncta) across different cell types. The plot displays a weighted distribution to emphasize the percentage of lysosomes larger than 0.5 μm^2^. **(B)** Density plots illustrating the number of lysosome puncta normalized to cell area across the indicated cell types. **(C)** Evaluation of lysosome proteolytic activity using a pan-cathepsin activity-based probe. **(D)** Representative images depict fluorescence intensity levels of the pan-cathepsin activity-based probe (Fire LUT scale). All quantifications were based on single-cell and single-object (puncta) segmentation using an Opera Phenix High-Content Microscope, involving *n* ≥ 300 cells and 3 independent experiments. All measurements were conducted simultaneously using DMEM media. Scale bar: 10 μm. **(E)** Evaluation of GAL-3 puncta in cells left untreated or treated with LLOMe (1 mM) for 10 min and 1 h. Representative images are included. All quantifications were based on single-cell and single-object (puncta) segmentation using an Opera Phenix High-Content Microscope, involving *n* ≥ 300 cells and 3 independent experiments. All measurements were conducted simultaneously using DMEM media. Scale bar: 10 μm. Plots were done using R Studio 2023.09.1.

The diverse compositions and activities of lysosomal enzymes observed in immune cells compared with those in conventional epithelial lines highlight the limitations of using universal models to capture lysosome function across cell types. While comparing lysosome properties across studies poses challenges, analyzing different cell types in parallel reveals significant variations in proteolytic activities, morphologies, and intracellular heterogeneity. Our analysis further reinforces this notion (Fig 1), demonstrating clear differences in lysosome morphology and activity between iPSC-derived macrophages, THP-1 cells, and commonly used epithelial cell lines. For example, macrophage lysosomes exhibit a considerably larger area compared with their epithelial counterparts, with further variations observed within epithelial cell lines themselves (Fig 1A). Lysosome number also show significant variation, with macrophages and RPE-1 cells harboring the highest numbers, and HEK-293T cells possessing the fewest (Fig 1B). Notably, functional differences in lysosome proteolytic activity are also evident, with macrophages displaying higher proteolytic capacity than epithelial cells (Fig 1C and 1D).

Adding another layer of complexity, lysosomes show heterogeneity not only across cell types but also within them, encompassing variations in membrane stability, acidification, and degradation capabilities. Recent findings challenge the paradigm that galectin-3 (GAL-3)-positive lysosomes were assumed to be destined for lysophagy [8]. These data suggest that GAL-3 is not a universal marker of membrane damage and highlight the possibility of lysosome repair circumventing GAL-3 involvement under specific conditions [9].

The dynamics of lysosome damage and repair, crucial for lysosome function, also showcase marked variations across cell types. Analyzing GAL-3 recruitment kinetics following lysosome damage in various cell types exemplifies this point (Fig 1E). While the potential role of other galectins in detecting lysosomal damage cannot be excluded [10], these findings emphasize the limitations of applying universal criteria and single markers to evaluate lysosome quality control mechanisms across diverse cell types. Each cell type might require context-specific evaluation due to distinct marker sets and mechanisms. This heterogeneity underscores the intrinsic complexity of cellular processes and necessitates context-specific evaluations when studying lysosome functions and related mechanisms. Furthermore, the discovery of several ESCRT-independent repair mechanisms alongside ESCRT-dependent pathways suggests a more intricate landscape of lysosome quality control than previously appreciated [3]. This raises intriguing questions. Does a specific repair mechanism dominate depending on cell type and stimuli? Can they coexist or target distinct lysosome populations? Identifying the specific molecular cues triggering these mechanisms remains a critical question.

Considering the observed heterogeneity among lysosome functions across different cell types, these findings collectively underscore the critical need for a refined approach to study lysosome biology. The marked differences in lysosome enzymes, morphology, and activity among cell lines and primary cells further highlight the inadequacy of a “one size fits all” approach in lysosome research. Consequently, we advocate for the use of multiplex profiling approaches as a concerted effort to identify and characterize lysosome states. By leveraging advanced techniques such as 3D quantitative live-cell imaging, this initiative aims to enhance our understanding of lysosome dynamics through context-specific evaluations.

To define the functions and dynamics of individual lysosomes (with the aim of understanding these states), approaches that shift away from lysosome population studies will be required. For example, although lysosome immunopurification is valuable in exploring cell-to-cell heterogeneity, its use is limited by its reliance on a single marker [11]. This dependence on marker expression, which can vary by lysosome type, cell type, and condition, hinders its accuracy in capturing individual lysosome diversity, highlighting the need to reevaluate not only our definition of a lysosome (including marker selection) but also our understanding of transient states such as repaired lysosomes, lysophagy-targeted lysosomes, and secretory lysosomes.

The future of lysosome research is bright. Advances in molecular tools capable of monitoring lysosome properties at single-organelle resolution offer a promising avenue for transcending previous limitations. The advent of novel reporters and probes capable of elucidating functional lysosomal attributes, such as proteolytic activity and ion concentration, at the level of individual organelles [12] and imaging techniques for tracking single organelles, in conjunction with specific markers and reporters, offer a potent strategy for unveiling the functional heterogeneity of lysosomes and providing real-time, dynamic insights into single lysosome function. They also allow for the early detection of subtle lysosome changes, which may precede the manifestation of lysosome-related diseases.

Given the complexities and inherent variability in lysosome functions across different cellular contexts, we believe the introduction of the lysosome states framework represents a significant leap forward in our understanding of lysosome dynamics and function. With each lysosome potentially serving as a sentinel of cellular health, the implications for diagnostics and treatment are substantial, charting a transformative path for future research in cell biology and pathology.
